# Evaluation of GnRH antagonist pretreatment before ovarian stimulation in a GnRH antagonist protocol in normal ovulatory women undergoing IVF/ICSI: a randomized controlled trial

**DOI:** 10.1186/s12958-021-00836-8

**Published:** 2021-10-12

**Authors:** Yisheng Zhang, Liling Liu, Jie Qin, Hongyi Huang, Lintao Xue, Shikai Wang, Weihong Tan

**Affiliations:** Department of Reproductive Medicine and Genetics Center, The People’s Hospital of Guangxi Zhuang Autonomous Region, 6 Taoyuan Road, Nanning, 530021 Guangxi China

**Keywords:** GnRH antagonist, Pretreatment, Synchronization, Controlled ovarian stimulation, Pregnancy outcome

## Abstract

**Background:**

Synchronization of follicles is key to improving ovulation stimulation with the gonadotropin-releasing hormone (GnRH) antagonist protocol. GnRH antagonist administration in the early follicular phase can quickly decrease gonadotrophin (Gn) levels and achieve downregulation before stimulation, which may improves synchronization. A previous small randomized controlled study (RCT) showed that pretreatment with a GnRH antagonist for 3 days before stimulation may increase oocyte retrieval but cannot increase the pregnancy rate. This study investigated whether the GnRH antagonist pretreatment protocol in ovulatory women can increase the synchronization of follicles and pregnancy outcomes compared with the conventional GnRH antagonist protocol.

**Methods:**

This RCT included 136 normal ovulatory women undergoing in vitro fertilization (IVF)/intracytoplasmic sperm injection (ICSI). Both groups were treated with recombinant follicle-stimulating hormone (r-FSH) and a flexible GnRH antagonist protocol. The women were randomized into two equal groups with or without GnRH antagonist administration from day 2 of the menstrual cycle for 3 days before stimulation. Our primary outcome was the number of retrieved oocytes. Secondary outcomes included the pregnancy rate and live birth rate.

**Results:**

Both groups had similar baseline characteristics. The number of retrieved oocytes in the study group was comparable to that in the control group (9.5 [8.0–13.0] vs. 11.0 [7.0–14.8], *P* = 0.469). There was no significant difference in the follicle size. The fertilization rate, number of good-quality embryos, implantation rate, pregnancy rate, ongoing pregnancy rate, live birth rate per embryonic transfer cycle, and miscarriage rate were similar between the two groups.

**Conclusion:**

This large RCT analysed GnRH antagonist pretreatment with the GnRH antagonist protocol applied to normal ovulatory women undergoing IVF/ICSI. The number of retrieved oocytes and pregnancy outcomes did not significantly vary.

**Trial registration:**

Chinese Clinical Trial Registry, ChiCTR1800019730. Registered 26 November 2018.

**Supplementary Information:**

The online version contains supplementary material available at 10.1186/s12958-021-00836-8.

## Background

With the development of the gonadotropin-releasing hormone (GnRH) antagonist protocol in theory and application, GnRH antagonists have become widely used tools for controlled ovarian stimulation (COS) cycles because of its advantages, including its shorter duration of stimulation, low cost, and low incidence of ovarian hyperstimulation syndrome (OHSS) [1–3]. It has been recognized as being practical and cost-effective for patients with high or poor ovarian responses [4]. Meta-analyses [4] have demonstrated similar live birth rates (LBRs) between the long GnRH agonist protocol and the GnRH antagonist protocol; therefore, the 2019 European Society of Human Reproduction and Embryology (ESHRE) guidelines for COS [5] recommend that the GnRH antagonist protocol be used in normal responders. However, the literature used to formulate these guidelines includes few randomized controlled trials (RCTs) on the LBR. Additionally, whether the GnRH antagonist protocol is applicable or beneficial to women with a normal response in terms of the clinical outcome has been controversial [3, 4].

As the GnRH antagonist protocol lacks the process of pituitary downregulation [6], the endogenous follicle-stimulating hormone (FSH) level is not inhibited, and the transient increase in endogenous FSH during the luteal-follicular transition recruits early antral follicles, thereby affecting the synchronization of follicles [7, 8]. Previous studies have proposed various pretreatment methods, with little success being observed in terms of solving the problem of follicular asynchrony. Studies have shown that pretreatment with oestradiol (E2) or progesterone (P) during the luteal phase increases the number of retrieved oocytes, as well as the clinical pregnancy rate and the LBR [9, 10]. Some studies have shown that pretreatment with the oral contraceptive pill (OCP) in the previous menstrual cycle may reduce the ongoing pregnancy rate (OPR) and LBR of the GnRH antagonist protocol [10]; therefore, the routine use of oral hormone pretreatment in an antagonist protocol may not be recommended.

It is assumed that the administration of the GnRH antagonist at the early stage of follicular development can quickly reduce the Gn level and achieve immediate- and short-term pituitary downregulation before the initiation of Gn stimulation. This process is conceptually similar to the long agonist protocol but maintains the advantages of the GnRH antagonist protocol. Based on this principle, pretreatment with a GnRH antagonist in the early follicular phase may improve follicular synchronization [11]. It appears that the GnRH antagonist pretreatment in the suggested GnRH antagonist protocol is feasible in the assisted-reproductive technology (ART) setting. The main benefit of this protocol is that all of the treatments are performed in the same cycle, thus making the process patient-friendly and short. One RCT [11] showed that for normal responders, pretreatment with a GnRH antagonist for 3 days before the start of ovarian stimulation could increase the number of mature oocytes and the fertilization rate. Another RCT [12] showed that the number of oocytes after antagonist pretreatment tended to increase, although the increase was not statistically significant. The sample size was small in both of these RCTs, and the conclusions are inconsistent. Therefore, whether pretreatment with a GnRH antagonist before the initiation of ovarian stimulation in patients with a normal ovarian reserve helps follicular synchronization and improves pregnancy outcomes remains to be investigated in larger studies.

In this context, we increased the sample size and hypothesized that GnRH antagonist pretreatment before the start of ovarian stimulation in the antagonist protocol can increase the synchronization of follicles. Our study aimed to conform  whether 3 days of GnRH antagonist pretreatment before ovarian stimulation could increase the number of retrieved oocytes and improve the pregnancy outcomes of ovulatory women undergoing in vitro fertilization (IVF)/intracytoplasmic sperm injection (ICSI) treatment.

## Methods

### Study design

**Fig. 1:**
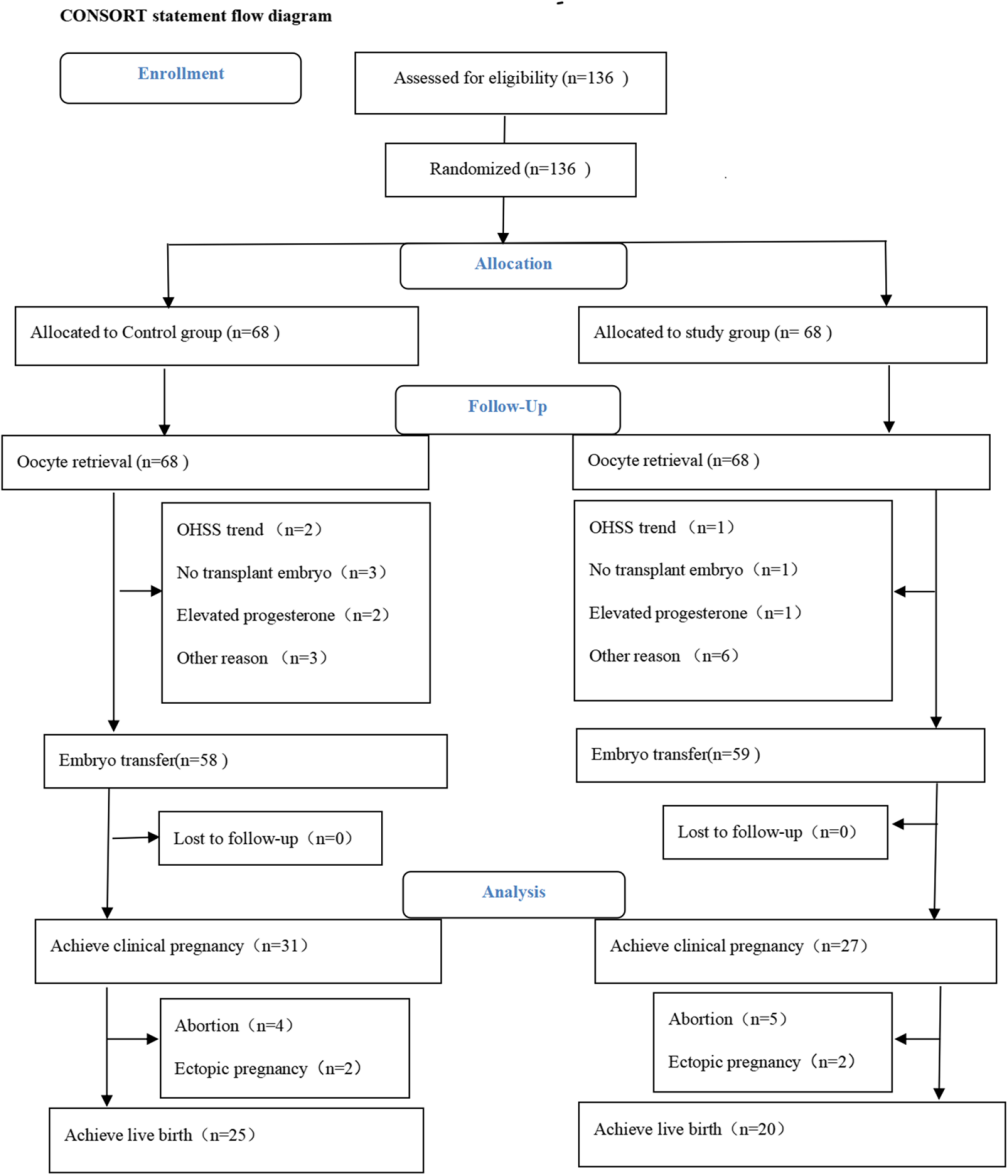
Flow diagram

Between December 2018 and March 2020, we prospectively recruited 136 normal ovulatory women under 40 years of age who underwent their first or second IVF/ICSI cycle at the Reproductive Medicine and Genetics Center of the People’s Hospital of Guangxi Zhuang Autonomous Region. On day 2 of the menstrual cycle, we randomly assigned the eligible patients to either the pretreatment GnRH antagonist protocol group (the study group) or the conventional GnRH antagonist protocol group (the control group) at a ratio of 1:1. All of the study participants were treated using a flexible GnRH antagonist protocol (Fig. 1).

### Recruitment and randomization

This was a prospective open RCT. As a placebo with the same dosage form and shape as a GnRH antagonist was not available, this trial could not be performed as a double-blind trial, which would include blinding of the oocyte retrievers, embryologists, statisticians, and hormone detection personnel involved in the study. With the randomized block design method, random numbers were generated using the Statistical Package for Social Sciences (SPSS, version 23.0) software and ordered from 1 to 136. The group was divided into 34 block groups, according to every four digits, in which one half comprised the study group and one half comprised the control group. We used a computer-generated random list for randomization. We created a series of consecutively numbered and opaque envelopes to seal the grouping details and hide them from the recruiting doctor. These envelopes were opened only when patients met the inclusion criteria.

We enrolled patients who met the group’s inclusion criteria, according to the sequence of medical treatment. All of the patients who participated signed informed consent forms, and each patient participated in this trial only once. We registered the RCT with the Chinese Clinical Trial Registry (registration number: ChiCTR1800019730), and the ethics committee of the People’s Hospital of Guangxi Zhuang Autonomous Region approved the trial.

### Study population

All consecutive women who underwent their first or second cycle of IVF/ICSI were included, and the first cycle included only normal responders.

The study inclusion criteria were as follows: age < 40 years; anti-Mullerian hormone (AMH) ≥1.2 ng/ml; antral follicle count (AFC) > 7; regular menstrual cycles over the 3 months before the study (25–35 days in duration); and a basal serum FSH concentration lower than 12 IU/L.

The exclusion criteria were as follows: endometriosis grade III to IV (American Fertility Society classification of endometriosis [13]); adenomyosis; diagnoses of polycystic ovary syndrome (PCOS) [14]; decreased ovarian reserve function (FSH > 12 U/L or AFC < 8 or AMH < 1.1 ng/ml) or a poor ovarian response [15], as defined by less than four oocytes being retrieved in a previous IVF or ICSI cycle; body mass index (BMI) > 30 kg/m^2^; severe male oligospermia or obstructive azoospermia; and use of hormone therapy within the 3 months before the study.

### Protocols

**Fig. 2:**
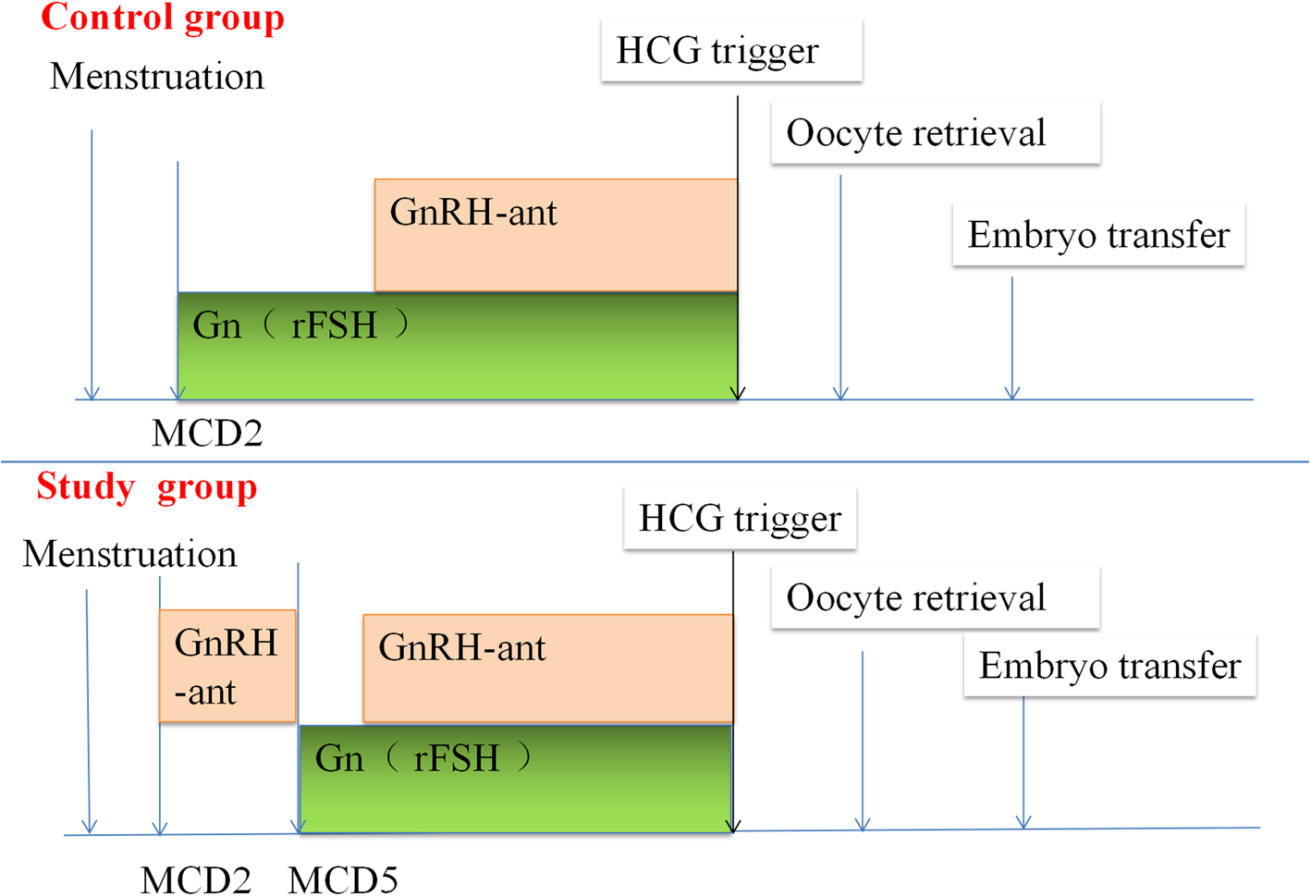
Protocols of the two groups. Note: *MCD*–menstrual cycle day; *GnRH-ant*–GnRH antagonist. In the control group, ovarian stimulation with Gn was initiated on day 2 of the menstrual cycle. In the study group, ovarian stimulation was initiated after 3 days of GnRH antagonist pretreatment

A baseline ultrasound examination and serum sex hormone analysis were performed on menstrual cycle day two and after the completion of GnRH antagonist pretreatment to determine the absence of ovarian cysts or lead follicles > 10 mm. In the conventional antagonist protocol (control group), ovarian stimulation with Gn was initiated on day 2 of the menstrual cycle. In the pretreatment GnRH antagonist protocol (study group), ovarian stimulation was initiated after 3 days of GnRH antagonist pretreatment (Cetrotide®, 0.25 mg cetrorelix acetate, Serono, Inc.). In both protocols, 150–225 IU of recombinant FSH (rFSH) (Gonal - F®, Serono Laboratories Ltd., Geneva, Switzerland) was used for ovarian stimulation. The dose of rFSH could be adjusted according to the patient’s response after ovarian stimulation for 3–4 days. In both groups, when the follicle size was ≥12 mm or the luteinizing hormone (LH) level was > 10 IU/ml, the GnRH antagonist was given at 0.25 mg/day until the human chorionic gonadotropin (HCG) trigger day. When the diameter of the two dominant follicles increased to 18 mm or more, or when the diameter of the three dominant follicles increased to 17 mm, we triggered ovulation with 250 μg of recombinant human chorionic gonadotropin (r-HCG) (Ovitrelle®, 250 μg/0.5 ml, Merck, Serono, Inc.). The serum E2, LH, and P levels were also considered in the decision to trigger ovulation. Thirty-six to thirty-eight hours after the trigger event, specialized physicians retrieved the oocytes. Two embryologists were assigned to perform the oocyte examinations. The embryo was cultured until day three or day five and then transplanted by specialized physicians. The follow-up nurse recorded the results of the follow-up examinations and the reasons for any losses (Fig. 2) [3].

### Outcome measures

The number of retrieved oocytes was the primary outcome of our study. The secondary outcomes were the HCG positive rate, clinical pregnancy rate (CLR) per embryo transfer (ET) cycle (defined as the presence of one or more gestational sacs upon transvaginal ultrasound, including an ectopic pregnancy) [16], OPR per ET cycle (a pregnancy beyond 12 weeks’ gestation), and LBR per ET cycle (defined as the delivery of a live-born infant after ≥28 weeks of gestation) [17]. Furthermore, we evaluated the following secondary adverse safety and pregnancy outcomes among the two study groups: the incidence of moderate to severe OHSS (according to the criteria proposed by Golan and Weissman [2009]) [18] and miscarriage rate, which was defined as foetal loss before the 28th week of gestation [17]. We used the follicular output rate (FORT) [19] to reflect the effect of follicular synchronization. The formula for calculating the FORT in this study was the number of follicles with a diameter of 14–22-mm on the HCG trigger day divided by the number of follicles with a diameter of 3–8-mm on the cycle entry day.

### Statistical considerations

#### Sample size calculation

This was an open prospective RCT. We calculated the sample size by using a power analysis and sample size (PASS, version 11.0). We estimated the sample size based on the results of the retrieved oocytes from one published RCT, the actual number of retrieved oocytes at our centre, the mean number of retrieved oocytes in the control group (*n* = 7), the mean number of retrieved oocytes in the study group (*n* = 10), and a standard deviation = 5. We estimated the dropout rate to be 15% among patients who had cancelled cycles prior to oocyte retrieval for various reasons, such as no oocyte retrieval or oocyte acquisition. The application of a clinically justified dropout rate of 15% yielded a total number of 136 patients to be randomized. To achieve 80% power by using a 1:1 randomization ratio, each study group required 68 subjects (136 patients in total).

### Statistical analysis

The data are reported in accordance with the 2010 Consolidated Standards of Reporting Trials (CONSORT) clinical trial guidelines [20]. We used IBM SPSS, version 23.0, software for the statistical analysis. Normally distributed data are represented by the mean and standard deviation (SD), and skewed data are described as the median and interquartile range (IQR). We used the chi-square test or Fisher’s exact test (when appropriate) to make statistical inferences on the qualitative data. In contrast, we used the t-test or the Mann-Whitney test to compare the continuous variables, as required. A probability (P) value < 0.05 indicated that the difference between the two groups was statistically significant.

## Results

### Study flow-chart and baseline characteristics

This trial included 136 infertile normal ovulatory women undergoing their first or second cycle of IVF/ICSI. After randomization, there were 68 women in each group. The patient demographics and clinical characteristics are presented in Table 1. Baseline data, such as age and AFC, were similar between the two groups. Table 1 presents the ovulation stimulation parameters of both protocols. The stimulation length (8.6 ± 1.2 days vs. 8.8 ± 1.6 days) and total Gn amount (1813.6 ± 398.2 IU vs. 1766.4 ± 415.8 IU) were similar between the control and study groups. There was no significant difference in the amount of GnRH antagonist added after stimulation between the control and study groups (1.08 ± 0.3 vs. 1.09 ± 0.3, *P* = 0.830); however, there was a significant difference in the total amount of GnRH antagonist between the control and study groups (1.1 ± 0.3 vs. 1.8 ± 0.3, *P* < 0.001).

**Table 1:** Main subject and cycle characteristics

Variables	study group (*n* = 68)	control group (*n* = 68)	*p-* value
Age (years), mean ± SD	33.0 ± 3.4	32.1 ± 3.8	0.152
BMI (kg/m^2^), mean ± SD	21.5 ± 3.5	21.4 ± 3.0	0.812
Infertility duration (years), median (IQR)	3.0(2.0–5.0)	3.3(2.0–6.0)	0.477
AFC(n), median (IQR)	13.0(11.0–17.0)	12.5(10.0–17.0)	0.605
AMH(ng/ml), median (IQR)	2.7(1.9–4.0)	2.7(1.5–4.3)	0.855
Diagnosis of infertility			0.377
Tubal factor,n(%)	39/68(57.4)	48/68(70.6)	
Unexplained infertility,n(%)	4/68(5.9)	5/68(7.4)	
Male factor, n(%)	10/68(14.7)	8/68(11.8)	
Combining male and female factors, n(%)	10/68(14.7)	5/68(7.4)	
Three AIH failure history,n(%)	5/68(7.4)	2/68(2.9)	
Stimulation length (day), mean ± SD	8.8 ± 1.6	8.6 ± 1.2	0.629
Gn amount(IU), mean ± SD	1766.4 ± 415.8	1813.6 ± 398.2	0.500
GnRH-ant amount after initiating GN	1.09 ± 0.3	1.08 ± 0.3	0.830
Total GnRH-ant amount(mg), mean ± SD	1.8 ± 0.3	1.1 ± 0.3	< 0.001

Note: *AMH* anti-Mullerian hormone, *AFC* antral follicle count, *BMI* body mass index, *IQR* interquartile range, *SD* standard deviation, *AIH* artificial insemination with husband semen, *GnRH-ant* GnRH antagonist

### Efficiency outcome measures and pregnancy outcomes

There was also no statistically significant difference in the number of retrieved oocytes (11.0 [7.0–14.8] vs. 9.5 [8.0–13.0]), number of mature oocytes (9 [5.3–12.0] vs. 7.0 [6.0–11.0]), FORT [19] (80.4% [54.0–100%] vs. 77.4% [53.5–100%]), number of two pro-nuclei (2PN) oocytes retrieved (6.5 [4.0–9.0] vs. 6.0 [4.0–8.0]), number of good-quality embryos (2.0 [1.0–4.0] vs. 2.0 [1.0–4.0]), or number of frozen embryos (2.0 [1.0–4.0] vs. 2.0 [0.0–4.0]) between the control and study groups (Table 2). The number of transferred embryos was similar in the two groups (Table 3). Although the implantation rate, HCG positive rate, clinical pregnancy rate, OPR, and live birth rate per ET cycle were lower in the study group than in the control group, the differences were not statistically significant (Table 3).

**Table 2:** Stimulation characteristics and embryological data

Variable	study group	control group	*p*- value
No. of follicles ≥14 mm at trigger day	8.0(6.0–11.0)	9.0(7.0–11.0)	0.568
Follicle output rate ^b^(%)	77.4(53.5–100.0)	80.4(54.0–100.0)	0.764
No. of oocytes retrieved (n)	9.5(8.0–13.0)	11.0(7.0–14.8)	0.469
No. of MII oocytes^c^(n)	7.0(6.0–11.0)	9(5.3–12.0)	0.485
MII oocyte rate(%)	80.2(71.4–100.0)	82.8(72.8–100.0)	0.991
No. of 2PN oocytes (n)	6.0(4.0–8.0)	6.5(4.0–9.0)	0.365
No. of transferable embryos (n)	5.5(4.0–7.8)	6.0(4.0–8.0)	0.236
No. of good quality embryos(n)^d^	2.0(1.0–4.0)	2.0(1.0–4.0)	0.708
No. of frozen embryos (n)	2.0(0.0–4.0)	2.0(1.0–4.0)	0.502

Note: Data are presented as the median and interquartile range (IQR)

^a^Control group used as a reference

^b^Follicular output rate determined by the ratio of the preovulatory follicle (14–22 mm) count on the HCG trigger day ×100/the small antral follicle (3–8 mm) count at baseline,

^c^MII-metaphase II

^d^Good-quality embryos included day-3 and day-5/6 high-quality embryos (according to our centre’s quality embryo scoring standards, day-3 embryos that were considered were grade 1–2 with 7–9 blastomeres and < 20% fragmentation [21]; blastocysts that were considered were at least at expansion stage 3, had an inner cell mass score of A or B, and had a trophectoderm score of A or B on day 5)

**Table 3:** Clinical outcomes and complications

Variable	study group	control group	*p*- value
No.of ET cycle (n)	59	58	–
ET cycle cancellation rates (n, %)	9/68(13.2)	10/68(14.7)	0.805
No.of Embryos transfered (n), mean ± SD	1.9 ± 0.3	1.9 ± 0.4	0.550
Implantation rate n,(%)	37/112(33.0)	39/108(36.1)	0.632
**Pregnancy**			
Biochemical pregnancy rate per ET cycle n,(%)	36/59(61.0)	35/58(60.3)	0.941
Clinical pregnancy rate per ET cycle, n, (%)	27/59(45.8)	31/58(53.4)	0.406
Ongoing pregnancy rate per ET cycle, n,(%)	20/59(33.9)	26/58(45.6)	0.306
Live-birth rate per ET cycle, n,(%)	20/59(33.9)	25/58(43.1)	0.197
**Pregnancy loss**			
Biochemical pregnancy, n,(%)	9/36(25.0)	4/35(11.4)	0.139
Abortion rate, n,(%)	5/27(18.5)	4/31(12.9)	0.556
Ectopic pregnancy rate, n,(%)	2/27(7.4)	2/31(6.5)	0.886
**Complication**			
Incidence of moderate-to-severe OHSS, n,(%)	1/68(1.5)	2/68(2.9)	0.559

Note: *ET* embryo  transfer, *SD* standard deviation

### Adverse safety and pregnancy outcome measures

Adverse pregnancy outcomes (specifically, biochemical pregnancy loss and clinical miscarriage) did not significantly vary between the two groups (Table 3). Moderate to severe OHSS also occurred at a similar rate in the study and control groups (1/68, 1.5%, vs. 2/68, 2.9%, respectively; *P* = 0.559).

### Changes in hormones at different points

The baseline endocrine levels in the two groups were similar (an additional table file shows this in more detail [see Additional Table 1]). When compared with those levels in the control group, the serum levels of FSH, E2, and P in the study group were significantly lower at the initiation of stimulation (*P* < 0.05). The LH level in the study group was higher than that in the control group on the day of antagonist addition, and the LH level increased in the study group compared to the control group (13.2% [9/68] vs. 7.4% [5/68], respectively). The increase in the P level (1.5% (1/68) vs. 0%) between the study group compared to the control group did not result in a statistically significant difference. On the HCG trigger day, the serum LH level was higher in the study group than in the control group (3.5 [2.5–6.1] vs. 2.1 [1.1–3.5] IU/L, respectively; *P* < 0.001), as was the E2 level (2659.0[1862.0-3359.2] vs. 2251.5[1421.5-3100.5], *P* = 0.069), but the serum progesterone level was similar in the two groups.

### Comparison of follicular development characteristics

As expected, the follicle sizes and variation coefficients (VCs) [22] of the two groups were similar on the cycle initiation day and the stimulation initiation day (an additional table file shows this in more detail [see Additional Table 2]). After the initiation of ovarian stimulation, we observed that the average follicle size in the study group on the day of antagonist addition was significantly lower than that in the control group (*P* < 0.05); however, the two groups had similar follicle variability, with an average follicle size ≥10 mm and the largest follicle size observed in both groups. On the HCG trigger day, the follicle size and VC of the two groups were similar.

## Discussion

Currently, this study is the largest RCT comparing the use of pretreatment with a GnRH antagonist to a standard flexible GnRH antagonist protocol in ovulatory women undergoing IVF/ICSI. The results show that compared with the traditional GnRH antagonist protocol, GnRH antagonist pretreatment before Gn initiation in the early follicular phase did not improve the synchronization of follicles in ovulatory women. The number of retrieved oocytes in the study group was comparable to that in the control group. There were no significant differences in the pregnancy outcomes.

Differences in the FSH threshold of follicles is the main reason for follicular asynchrony [8]. GnRH antagonist use in the early follicular phase can reduce the FSH level and may prevent the early recruitment of follicles. This study used the maximum diameter, average diameter, and VC of the follicles at each stage during COS to reflect follicular synchronization. The results showed that the diameter and variability of the antral follicles were the same. The maximum diameter, average diameter, and variability of the follicles on the day of antagonist addition and the HCG trigger day were not different between the two groups. Clearly, GnRH antagonist pretreatment in the early follicular phase did not achieve follicular synchronization, which is inconsistent with previous research results. One previous study [23] used antagonist pretreatment in the luteal phase to specify the difference in the follicle size. Nevertheless, the effect of antagonist pretreatment in the follicular phase on follicle size was not reported. In addition, we used the FORT to reflect the effect on follicular synchronization. The results showed that the FORT was not different between the two groups, which further indicates that the antagonist did not increase the synchronization of follicles after pretreatment. One of the main indicators used to measure the synchronization of follicles is the number of retrieved oocytes. In this study, the number of retrieved oocytes and the number of mature oocytes were not different between the two groups. There was no statistically significant difference in the number of available embryos, frozen embryos, or good-quality embryos. This is consistent with previous research results. One RCT [12] by Blockeel et al. recruited 69 women with a normal ovarian response. The number of retrieved oocytes after pretreatment with GnRH antagonist for 3 days on the first 3 days of menstruation showed an increasing trend (12.8 ± 7.8 vs. 9.9 ± 4.9), but with no significant difference. The case-control study by Veronique et al. [24] focusing on women < 35 years old with a normal ovarian response showed that pretreatment with GnRH antagonist for 3 days after menstrual days 2–4 did not affect the number of retrieved oocytes.

The timing and length of the use of antagonists are inconsistent; thus, the conclusions of the various studies may be incompatible. The RCT [11] by Younis JS et al. of women under 39 years old with a normal ovarian response showed that pretreatment with an antagonist (0.25 mg of GnRH antagonist per day for three consecutive days) starting on the first day of menstruation can increase the number of mature oocytes and the normal fertilization rate. Nevertheless, there was no comparison of pregnancy outcomes in their study. The reason for this effect may be that the FSH level begins to rise on the first day of menstruation, and antagonists can prevent the early recruitment of follicles caused by the increase in FSH. In our study, pretreatment with an antagonist was begun on the second day of menstruation. At this time, the follicles may have already been recruited. Even when using the antagonist, follicular recruitment cannot be reversed, and the asynchrony of follicles cannot be changed. As most of our patients lived at a far distance and could not see the doctor on the first day of menstruation, the antagonist could not be used on the first day.

The timing of antagonist addition may also affect the outcome. The antagonist protocol has a fixed and flexible protocol. Our study used a flexible antagonist protocol. The maximum follicular diameter and E2 level on the day of antagonist addition of in the two groups were consistent with those reported in the literature, and the differences between the two groups were not statistically significant (Additional Tables 1 and 2) [25].

Each patient’s ovarian response to stimulation also affects the results of antagonist pretreatment [26]. A meta-analysis of the delayed initiation of antagonist pretreatment for low-response patients showed that this measure reduces the Gn dose and increases the clinical pregnancy rate. Studies have also found that pretreatment with antagonists in PCOS patients can increase the biochemical pregnancy rate and significantly reduce the incidence of OHSS [27]. Patients with PCOS and poor responders were excluded from the study The influence of different ovarian reactions on the results can be excluded, thus making the research data more reliable.

In this study, the Gn stimulation days in the two groups were similar. Although the LH level in the study group was higher than that in the control group on the day of antagonist addition, the increase in the P level did not result in a significant difference between the two groups, which is consistent with previous results in the literature [28]. The E2 level and LH level on the HCG trigger day were higher in the treatment group than in the control group. The reason for this effect may be that even if pretreatment with GnRH antagonist for 3 days did not achieve the purpose of inhibiting follicular recruitment, the follicles began to develop after delayed initiation of stimulation. At the time of the HCG trigger, the cycle is in the late follicular phase, and LH receptors are exclusively expressed on granulosa cells; moreover, higher LH levels may cause more oestrogen production [29]. However, the deleterious effect of high E2 levels on endometrial receptivity has been controversial [30]. There was no statistically significant difference in the clinical pregnancy rate and OPR between the two groups in our study, which is consistent with previous research showing similar findings [12, 31].

The ultimate goal of ART is a live birth; namely, a healthy baby with one ovulation induction. Therefore, as a research indicator, the LBR can better demonstrate the efficacy of ART. There has been no analysis of the LBR in previous studies. Therefore, we calculated the LBR in this study. The results showed no significant difference in the LBR between the two groups. We observed that the pretreatment did not affect the LBR. Additionally, we compared the incidence of moderate to severe OHSS, and the results showed that the incidence of OHSS was not increased after pretreatment.

In summary, pretreatment with antagonists has no significant impact on the pregnancy outcomes of ART and does not increase the risk of OHSS. Additionally, this approach can be used as a flexible protocol.

### Strengths and limitations

#### Advantages

First, our research sample size was large. The sample size of this study was calculated based on the number of retrieved oocytes in previous reports in the literature and our actual average number of retrieved oocytes. After our calculation, the sample size was 132 individuals. The sample size of a similar previous study in 2011 was only 69 individuals. Our sample size was significantly higher, providing sufficient testing power, and the results are more convincing. Second, we examined additional indicators, such as the follicle diameter at each node, the follicle VC, and the FORT, in order to reflect the synchronization of follicles in multiple ways, which is a more objective approach than using only the number of retrieved oocytes. Third, we increased the LBR per transplant cycle to quantify ART outcomes, thus making the research data more complete.

#### Limitations

First, as our patients could not see the doctor on the first day of menstruation, they could only use GnRH antagonist pretreatment on the second day of menstruation. Second, patients in the study group used the GnRH antagonist for three more days than patients in the control group; however, this difference neither increased the number of retrieved oocytes nor improved the pregnancy outcomes. Instead, it increased the number of visits and treatment costs for these patients.

This RCT was a single-centre study. We hope to confirm this conclusion in a future multicentre  RCT with a larger sample. Alternatively, whether pretreatment with a GnRH antagonist throughout ovarian stimulation improves the outcomes of patients with unsynchronized follicular development remains unclear.

## Conclusion

For normal ovulatory women, pretreatment with GnRH antagonists for 3 days before the start of ovarian stimulation in the early stage of follicular development neither increases follicular synchronization nor improves the clinical outcomes of IVF-ET.

## Supplementary Information

**Additional file 1: Additional Table 1.** Changes in hormones at different time points.

**Additional file 2: Additional Table 2.** Comparison of follicular development characteristics.

## Data Availability

The datasets that were used and/or analysed during the current study are available from the corresponding author upon reasonable request.

## Abbreviations

GnRH: Gonadotropin releasing hormone
IVF: In Vitro Fertilization
ICSI: Intracytoplasmic Sperm Injection
RCT: Randomized controlled trial
Gn: Gonadotrophin
PCOS: Polycystic ovary syndrome
POR: Poor ovarian response
FSH: Follicle stimulating hormone
r-FSH: Recombinant FSH
ET: Embryo transfer
E2: Estradiol
LH: Luteinizing hormone
COS: Controlled ovarian stimulation
OHSS: Ovarian hyperstimulation syndrome
ESHR: European Society of Human Reproduction and Embryology
P: Progesterone
OCP: Oral contraceptive pill
AMH: Anti-Mullerian hormone
AFC: Antral follicle count
BMI: Body mass index
HCG: Human chorionic gonadotropin
r-HCG: Recombinant human chorionic gonadotropin
MCD: Menstrual cycle day
GnRH-ant: GnRH antagonist
CLR: Clinical pregnancy rate
OPR: Ongoing pregnancy rate
LBR: Live birth rate
PASS: Power Analysis and Sample Size
CONSORT: Consolidated Standards of Reporting Trials
SPSS: Statistical Package for Social Sciences
SD: Standard deviation
IQR: Inter quartile range
CI: Confidence interval
RDs: Risk differentials
IU: International unit
AIH: Artificial insemination with husband’s semen
FORT: Follicle output rate
2PN: Two pronuclei
VC: Variation Coefficient
GC: Granulosa cells
ART: Assisted reproductive technology
